# Interprofessional collaboration in the breast cancer unit: how do healthcare workers see it?

**DOI:** 10.1186/s12905-022-01818-7

**Published:** 2022-06-13

**Authors:** Dea Anita Ariani Kurniasih, Elsa Pudji Setiawati, Ivan Surya Pradipta, Anas Subarnas

**Affiliations:** 1 Department of Pharmacology and Clinical Pharmacy, Faculty of Pharmacy, Universitas Padjadjaran, Raya Bandung Sumedang Km. 21, Hegarmanah, Jatinangor, Kabupaten Sumedang, 45363 Jawa Barat Indonesia; 2 Doctoral Program of Pharmacy, Faculty of Pharmacy, Universitas Padjadjaran, Sumedang, Indonesia; 3 Pharmacy Study Program, Akademi Farmasi YPF, Bandung, Indonesia; 4 Department of Public Health, Faculty of Medicine, Universitas Padjadjaran, Sumedang, Indonesia; 5 Drug Utilization and Pharmacoepidemiology Research Group, Center of Excellence in Higher Education for Pharmaceutical Care Innovation, Universitas Padjadjaran, Sumedang, Indonesia

**Keywords:** Health practitioners, Interprofessional collaboration, Indonesia, Qualitative

## Abstract

**Background:**

Interprofessional collaboration has an important role in health care for breast cancer patients who are undergoing treatment at the hospital. Interprofessional collaboration has been reported to provide significant benefits for patients. However, qualitative research on interprofessional collaboration in the breast cancer department is rarely done, therefore, a study was conducted to determine the perception of health practitioners about interprofessional collaboration in the breast care unit at a referral centre hospital in West Java, Indonesia.

**Methods:**

A qualitative study was carried out using in-depth interviews and focus group discussions (FGDs) with 15 healthcare personnel using total sampling. Participants were chosen among healthcare professionals who treat and in charge for outpatient breast cancer, but were not resident physicians. The FGD approach was used for nurses and pharmacists, and interviews were used for oncologists. The audio recordings of all interviews and FGDs were transcribed verbatim and evaluated using thematic analysis.

**Result:**

The findings were categorized into two categories to obtain health care workers' perspectives on interprofessional collaboration: (1) impediment factors: personality, lack of leadership, seniority, healthcare workers with double positions, the need for a clinical meeting, hospital bureaucracy, national health insurance implementation, issues with patients, hospital infrastructure, and evaluation and synchronisation; (2) existing supportive elements: effective cooperation, effective communication, clear job description, interpersonal relationships, Standard Operational Procedure (SOP) for cancer therapy, legality for inter-discipline cancer team, professional responsibility, integrated clinical pathway, patient centred care, and comprehensive health services.

**Conclusions:**

Interprofessional collaboration was seen positively by the respondents. However, there are several hurdles that must be overcome to apply interprofessional collaboration works effectively. The findings of this study can be used to build interprofessional collaborations targeted at enhancing quality health care in breast cancer units.

## Introduction

Breast cancer is the most frequently diagnosed type of cancer in females and the main cause of cancer death [1]. 154 countries have the disease as their most common cancer diagnosis, and it is the leading cause of cancer mortality in over 100 of those nations [2]. Cancer management is complex and needs several approaches in diagnosis and treatment such as surgery, systemic therapy (chemotherapy, immunotherapy, endocrine therapy) and radiotherapy. A multidisciplinary team should administer these diagnostic and therapeutic approaches, as part of integrated, patient-centred care [3]. With patients, families, caregivers, and communities, multiple health workers from all backgrounds collaborate to provide high-quality care. It enables health workers to work with everyone who can help accomplish local health goals. Collaboration among healthcare professionals enables the delivery of more complete care to patients, which contributes to enhanced treatment quality, a lower incidence of medical malpractice, shorter hospitalisation, and a lower death rate [4].

Interprofessional collaboration appears to be becoming increasingly important in the treatment of cancer patients, who require increased skill and expertise throughout the disease's progression: it is thus a matter of transforming healthcare services by fostering information-sharing and decision-making partnerships in order to place the patient at the centre of increasingly personalised and humanised healthcare pathways [5–7]. In the case of activities carried out by homogeneous professional groups in cancer treatment, interprofessional collaboration necessitates a rearrangement of traditional work and a shift in perspective. The transition from a monoprofessional to an interprofessional approach to healthcare is not a seamless process that can be taken for granted, but does involve some organisational and training changes [8].

However, the majority of health care workers do not have an accurate understanding of interprofessional collaborative practice [9]. Previous study showed that interprofessional collaboration in East Javan health centres is complicated and entangled at individual, organisational, and system levels where physicians are seen as leaders and decision makers in the traditional collaborative practice approach, which emphasises two-way communication [10]. According to Yani, there is a need to develop a consistent model of interprofessional collaboration, hospital policies that enable its implementation, information technology systems, and human resource development [11].

Unfortunately, qualitative studies on the practice of interprofessional collaboration in breast cancer units are limited. Therefore, we performed a study to assess healthcare practitioners' perspectives of interprofessional collaboration in a breast care unit at an Indonesian national referral centre hospital.

## Methods

### Study design

Between February and March 2021, we performed four in-depth interviews and four focus group meetings with health care professionals from several disciplines in a breast care unit, using a qualitative study approach (n = 15) with total sampling from a variety of backgrounds, ages, genders, and interprofessional collaborative experience. The inclusion criteria for this study were healthcare workers who treat outpatient breast cancer and were not resident physicians. Within the scope of this study, we took a qualitative approach. Professionals responsible for the treatment of breast cancer patients were selected for interviews and signed a consent form to participate in this research. Because of the workload of the oncologists, the interview took place in the oncology room and via video conference, and the FGDs took place in the head nurse's room. In-depth interviews are divided into three parts, namely opening, main, and closing questions, where the opening questions are describing participants experience in the breast care unit, participants’ thoughts on interprofessional collaboration in health care and what are their thoughts on the present practice in your workplace, and participants’ thoughts about the ideal interprofessional collaborative care looks like. Only the interviewee and interviewer were present at the interview location for in-depth interviews and focus group discussions. All of the interviews and FGDs were recorded on audio. During the interview and FGDs, field notes are utilised to keep track of essential details. Same questions were asked during in-depth interviews and FGDs. The main questions concern participants opinion about advantages of interprofessional collaborative care and giving instances from their day-to-day work/practice, participants view about which aspects will facilitate interprofessional collaboration in healthcare and participants’ belief about variables that would make interprofessional collaborative care more difficult. The final question asks what participants would do in their practice if they could change the system or become a policymaker for interprofessional collaborative care. There was no prior link between the researchers and the participants. Participants were made aware that the purpose of this study is to determine the current state of interprofessional collaboration in the breast cancer unit.

### Context and setting

The study was carried out in one of West Java's tertiary hospitals, which also served as a referral centre for breast cancer. In primary, secondary, and tertiary care, a patient-centred team approach is essential [12]. The interviews were done by DAAK as a PhD student with a hospital pharmacy background and interested in interprofessional collaboration research. DAAK has been trained in qualitative research methods such as in-depth interviews and FGDs.

Each participant signed an informed consent form. In-depth interviews and focus groups (FGDs) were used to conduct all of the interviews. When time allowed, in-depth interviews with specialists were undertaken, and focus groups with other healthcare workers were held. Three interviews for specialists, three groups of nurses, and one group of pharmacists were interviewed. The participants were purposefully grouped in uniprofessional groups in order to create a more suitable environment for expressing viewpoints.

### Information’s trustworthiness and credibility

Saturation occurred when no new information was received from participants and all healthcare providers who treated breast cancer outpatients were interviewed. In-depth interviews were audio-recorded, whereas FGDs were audio visually captured so that participant statements could be recognised during data processing. We provided the transcription findings to the participants without force in order for them to be corrected. Other sources, including documentation, regulations, and standard operating procedures, were also investigated in order to strengthen the reliability of the material.

### Data analysis

**Fig. 1:**
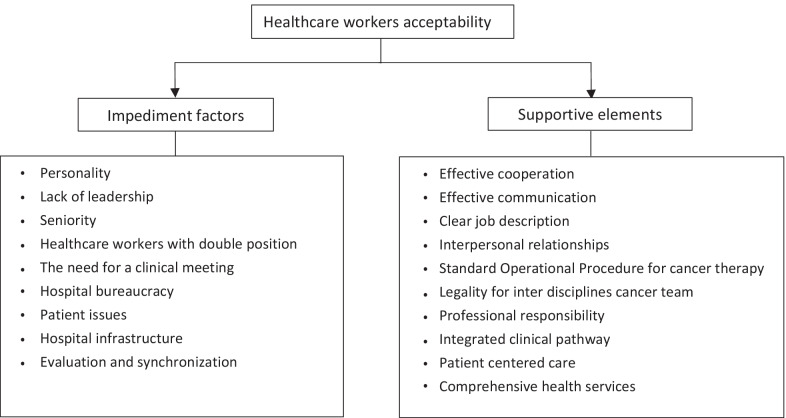
Healthcare workers acceptability regarding interprofessional collaboration

The transcripts were analysed using the thematic analysis method [13]. DAAK and EPS conducted an intermediate analysis, separately analysing the transcripts and using open coding to isolate meaningful quotations and concepts. The two researchers then compared and discussed their codes until they reached consensus, and then classified the detected concepts into subcategories and bigger categories. Finally, the team came to an agreement on a final set of primary categories and subcategories as seen in Fig. 1.

### Ethical considerations

The Research Ethics Committee Universitas Padjadjaran Bandung gave its approval to the project (number 882/UN6.KEP/EC/2020). All qualitative data has been encrypted and is accessible only to the principle investigator (DAAK). In the in-depth interviews and FGD transcriptions, as well as in any reports or publications that arose from the project, pseudonyms were employed.

## Results

We acquired consent in Bahasa Indonesia (Indonesian language) from 15 participants. There were no other local languages employed because all participants can communicate in Bahasa Indonesia, both orally and in writing. The demographics of the participants in the in-depth interviews and focus groups are summarised in Table 1. The majority of participants were male, with the biggest proportion between the ages of 45 and 54. Nurses are the most often interviewed health professionals, and up to 93.3% have expertise with interprofessional collaboration methods. As a result of the interviews and focus groups, two characteristics addressing the acceptability of interprofessional collaboration emerged, as seen in Fig. 1.

**Table 1:** Demographic characteristics of respondents in breast care unit (n = 15)

Variable	Total (n)	Percentage (%)
*Sex*		
Male	9	60.0
Female	6	40.0
*Age*		
25–29 year old	1	6.7
30–34 year old	0	0.0
35–39 year old	3	20.0
40–44 year old	2	13.3
45–54 year old	7	46.7
55–59 year old	2	13.3
*Health profession*		
Nurse	10	66.7
Oncologist	3	20.0
Pharmacist	2	13.3
*Work experience in the health care field*		
1–5 year	1	6.7
5–10 year	2	13.3
> 10 year	12	80
*Collaboration team*		
Palliative	3	18.8
Primary care	2	12.5
Burn unit	1	6.3
Oncology	6	37.5
Transplantation	1	6.3
Emergency	1	6.3
Surgery	2	12.5
*Current position in team*		
Team leader	1	7.7
Team member	12	92.3
*Interview duration, in minutes (mean, min–max)*		
FGD	18.70 (14–25)	
In-depth interview	18.30 (9–22)	

We identified two themes: (1) impediment factors and (2) supportive elements. The classification of themes and sub-themes is described in Fig. 1.

### Impediment factors

This study identified various impediments to interprofessional collaboration including personality, lack of leadership, seniority, healthcare workers with double position, the need for a clinical meeting, hospital bureaucracy, patient issues, hospital infrastructure, and evaluation and synchronisation.

#### Personality

‘*Professionals must recognize from the outset that cancer therapy necessitates a complete approach, therefore ego must be set aside*.’ – IV_P2_Oncologist

‘*That was the ego of each part, or the ego of the division*.’ – IV_P2_Oncologist.

Every interprofessional health worker must set aside ego for team success.

#### Lack of leadership

‘*In my opinion, we are still working together, just working together, working here and working there, in particular, maybe the leader itself has not coordinated with what's that ..*’ – IV_P3_Oncologist

‘*From the communication that is the most difficult, the issue is that the doctor is not always available*’ – FGDs_P15_Pharmacist

A leader is required to encourage the practice of interprofessional collaboration in daily work. Interaction with the leader for communication in interprofessional collaboration is occurring more frequently to discuss breast cancer outpatient cases in order to achieve treatment success.

#### Seniority

‘*Consider oneself a senior*’ – FGDs_P11_Nurse.

Seniority can have an impact on the work environment of team members in interprofessional collaboration.

#### Healthcare workers with double position

‘*Is it because I'm not paying attention here? There is a sense that the pharmacist is working at the same time*. ’ – FGDs_P14_Pharmacist

Due to limited human resources, several health workers hold multiple positions and do not focus on a single work unit.

#### The need for a clinical meeting

‘*Well, sometimes the implementation is still lacking because something is done in a clinical meeting*.’ – IV_P3_Oncologist

#### Hospital bureaucracy

‘*The point is that it has to be one voice, starting from the first time the patient comes to check the pain, then what is determined from there and how does it continue, what should the flow be*.’ – FGDs_P4_Nurse

#### Patient issues

‘*Like medicines that doctors recommend but are not approved by National Health Insurance, the patient continues to object to paying for his own expensive medicines*.’ – FGDs_P5_Nurse

‘*Patients don't have money for the treatment*’ – FGDs_P6_Nurse

Patients cannot afford to purchase medications outside of the national formulary, and the distance between their homes and health care facilities hinders patient care.

#### Hospital infrastructure

‘*Perhaps what has to be done is for the e-medical record to make it easier to comprehend professional writing or unclear instructions*. ’ – IV_P3_Oncologist

Improving hospital infrastructure is one way to ensure patient safety in treatment.

#### Evaluation and synchronization

‘*So, actually the format already exists, but maybe for the implementation it needs evaluation and what do you need to synchronise again?* – IV_P3_Oncologist

Interprofessional collaboration as a means of evaluating and developing quality services.

### Supportive elements

The following are some of the benefits that can be acquired in the breast cancer unit through interprofessional collaboration such as effective cooperation, effective communication, clear job description, interpersonal relationship, Standard Operational Procedures for cancer therapy, legality for inter-discipline cancer teams, professional responsibility, integrated clinical pathway, patient centred care, comprehensive health services.

#### Effective cooperation

‘*Between oncologist leader, nurses and pharmacists, we must always work together and communicate well so that there is no miscommunication between us officers and patients and other health workers*’ – IV_P10_Nurse

#### Effective communication

‘*Cooperation, coordination and clear job description explanations for everyone who is old or new*..’ – FGD_P11_Nurse

Communication and cooperation between health workers in interprofessional collaboration is needed to inform the health status of breast cancer patients. Each member of the interprofessional collaboration team has a distinct job to play.

#### Clear job description

‘W*e are from various professions but for coordination we already know each other's work, so now it's good for everyone to have their own job description.’* – FGD_P11_Nurse

#### Interpersonal relationship

‘*Interpersonal relations are influential. If, for example, the person has a good relationship, all that's left is to do something, it will be easier to communicate*.’ – IV_P1_Oncologist

Interpersonal interactions are maintained in interprofessional collaboration to sustain team dynamics.

#### Standard operational procedures for cancer therapy

‘*Not bad considering each has their own SOP*.’ – FGDs_P11_Nurse.

SOPs for cancer therapy (such as adjuvant breast carcinoma chemotherapy with FAC drugs and adjuvant breast carcinoma chemotherapy with paclitaxel and carboplatin drugs) that outline the duties and responsibilities of health professionals in cancer therapy have also been demonstrated to be a helpful element for interprofessional collaboration.

#### Legality for inter disciplines cancer team

‘*Continue with the cancer team, because the cancer team is activated, everyone is invited to collaborate there, there is no problem, we already have it, does not mean not have*’ – IV_P2_Oncologist

The need for interprofessional collaboration teams in the management of breast cancer patients is obvious in order to improve patient clinical outcomes. Each health worker's participation will make a beneficial difference in the patient's treatment. The necessity for legality from management for interprofessional collaboration is required as a guide for the team's operation.

#### Professional responsibility

‘*So indeed, the role of the pharmacist here is doctor's prescription review. We can ensure that the dose given to the patient is appropriate*.’ – FGDs_P14_Pharmacist

A health worker's professional obligation in interprofessional collaboration is necessary to ensure that they have the capacity and skills to manage instances of breast cancer patients.

#### Integrated clinical pathway

‘*The patient management protocol was formulated by the doctor himself, and now it is integrated with the clinical pathway*.’ – IV_P1_Oncologist

A hospital's medical oncology and hematology division created an integrated clinical pathway, which is a treatment guideline for cancer patients, in order to ensure patients' services.

#### Patient centred care

‘*The approach to patients and their families must be prioritised, so we can't choose whether these are rich people, these are poor people, it's not like that anymore*.’ – FGDs_P13_Nurse

Patients and their family will be assisted in treatment therapy so that patients understand the risks and benefits.

‘*If you collaborate more, you will definitely have a better outcome because our goal is patient-centred care, so patients can get the most out of it*.’ – IV_P3_Oncologist

Interprofessional collaboration allows health professionals to contribute professionally to better patient outcomes.

#### Comprehensive health services

‘*The advantage is that patient management is not compartmentalised, so that everything is done comprehensively*.’ – IV_P2_Oncologist

Participants believe that patients will feel more at ease with their therapy if they have access to a variety of services.

## Discussion

We discovered that the majority of the health care workers had prior experience with interprofessional collaboration, as shown by their participation in an interprofessional team that included a breast cancer team. This is also demonstrated by the 2015 hospital director's directive establishing a multidiscipline cancer management team [14]. The findings of this study contradict an earlier study indicating that the majority of healthcare professionals do not yet have an appropriate view of interprofessional collaborative practice [9].

According to our findings, there are various barriers to implementing interprofessional collaboration practices in the breast cancer unit, including personality issues, with interpersonal/interprofessional interactions being one of the contributing factors [15]. Our research indicates that there is still a weakness in unit cancer teams, namely in terms of leadership. This is consistent with Soemantri's research, which found that leadership is an important role in the success of interprofessional collaborative practices [15]. In interprofessional collaboration, leaders develop into frontline managers who serve as the team's motor. In order to achieve the overall goals for the services, frontline managers must encourage individual and collective efforts [16]. However, it takes a professional leader to become the engine of the organisation, organising and coordinating interprofessional collaboration, and guiding team development on a regular basis [17]. While interprofessional collaborative care validates a position on the team for a number of recognised professions, physicians continue to be the primary gatekeepers of patient access to a variety of other health professionals and services [6]. Developing interprofessional collaboration is not solely a managerial or policymaking responsibility; it also demands the active participation of professionals [18]. Another barrier discovered in this study is seniority, which determines which members of the team will become leaders based on the structure of the team hierarchy [19]. Due to the restricted quantity of human resources, the existence of many roles for a given professional also becomes an impediment that has an effect on the practice of interprofessional collaboration [20]. Our research further indicates that clinical meeting to discuss patient therapy are required. Sharing leadership could be achieved by having all practitioners participate in rounds or having equal say in patient talks [21]. Interprofessional collaboration facilitates the process of patient therapy [22–24]. According to a recent study, healthcare professionals must be trained on the importance of interdisciplinary collaboration, and cohesion of the group [25] in order to ensure medication safety [26], enhanced clinical decision-making, greater patient coordination, more evidence-based treatment decisions, and overall treatment quality [27]. According to Wulandari's research, another issue that hinders the practice of interprofessional collaboration is the presence of a complex bureaucracy [28]. Unavailability of electronic medical records that might facilitate and bridge collaboration between health workers from different specialties is an issue that complicates hospital infrastructure [29]. In order to enhance the quality of services, the assessment and synchronization phases must be conducted on a platform for interprofessional collaboration that contributes to a better understanding of health professionals [30, 31].

Our study found that there are elements that foster interprofessional collaboration in the breast cancer unit, giving health workers the confidence to collaborate in teams to improve health care for patients. It will be easier for health personnel to work with patients if they have complete documents. This is consistent with prior studies, which found that teamwork is more effective when professionals and patients collaborate, professionals coordinate, and teams develop over time where there is a requirement for consistency and regularity in the collaboration of all participants [5, 32].

Collaboration involves excellent communication [33, 34] and cooperation [35–37]. Another thing that helps is having clear job descriptions. They are both an organizational method that must be implemented and a talent that each member of the team must possess in order for interprofessional collaboration to be successful [38]. Interpersonal interactions between professionals also contribute to efficient interprofessional collaboration, which can be achieved by having the same goals, placing the patient first, understanding each other, and having mutual trust [39]. SOPs were also discovered to be a helpful feature in interprofessional teamwork. SOPs for breast cancer therapy can clarify what each health worker must do to assist a patient [19].

Legality is required as a supporting document to promote interprofessional collaboration activities in health care institutions [40]. Professional responsibility is one of the components of collaborative practice that contribute to the formation of an interprofessional team dedicated to achieving common goals in order to enhance patient outcomes [33, 41]. Clinical pathways, on the other hand, offer unique opportunities in interprofessional practice for designing and assessing patient-centred treatments [42]. Our study found that patient-centered approaches are being created to ensure that both patients' and providers' requirements and expectations are satisfied with regard to organized healthcare systems and infrastructure, which are essential to provide treatment that serves the needs of patients equitably [6]. Other findings suggested that better teamwork among multiple experts may be required to provide effective and comprehensive care [43].

However, while collaborative practice has gained widespread acceptance in healthcare, operationalising and quantifying this multidimensional notion in practice has proven challenging [21]. In addition, to collaborate effectively professionals need to have a positive dispositional humility that enables them to accurately judge themselves, to openly accept new ideas, to recognise others' contributions, and to cultivate compassion [44].

### Strength and limitation

Several of the study's limitations can be summarised as follows: This qualitative study examines the perspectives of health care professionals who treat breast cancer outpatients but has not yet interviewed hospital administrators about the unit's goal for interprofessional collaboration. This requires additional inquiry in order to provide a more comprehensive study. This study has used several approaches, such as triangulation and member check methods, to increase validity and reliability.

## Conclusion

The results of this study form the foundation for health workers' acceptance of interprofessional teamwork in the breast cancer unit. The future direction is when determining the existence of interprofessional collaboration aimed at improving patient clinical services, it is necessary to understand the factors that influence both in terms of strengths and weaknesses, so that it can be followed up on what steps should be taken for the team's sustainability and outcomes. It is also important to look at the elements that influence individuals, groups, and organisations.

## Data Availability

The related author's data storage contains all audio cassettes and typed transcripts. Due to ethical concerns, the datasets collected and/or analysed during this work are not publicly available; however, they are available from the corresponding author upon reasonable request, taking into account ethical concerns in the qualitative study.
